# Immune microenvironment of tumor-draining lymph nodes: insights for immunotherapy

**DOI:** 10.3389/fimmu.2025.1562797

**Published:** 2025-04-11

**Authors:** Jiahuan Wei, Daozhang Li, Haixia Long, Mei Han

**Affiliations:** ^1^ School of Pharmacy and Bioengineering, Chongqing University of Technology, Chongqing, China; ^2^ Institute of Cancer, Xinqiao Hospital, Third Military Medical University, Chongqing, China; ^3^ Department of Gynecologic Oncology, Chongqing University Cancer Hospital & Chongqing Cancer Institute & Chongqing Cancer Hospital, Chongqing, China

**Keywords:** tumor-draining lymph nodes, immune microenvironment, immunosuppressive cells, immunotherapy, PD-L1/PD-1, CD8^+^ T cells

## Abstract

Tumor-draining lymph nodes (TDLNs) play a crucial role in modulating tumor immune responses and influencing the efficacy of immunotherapy. However, our current understanding of the microenvironment within these lymph nodes remains limited. Tumors not only impair the anti-tumor activity of CD8^+^ T cells by creating an immunosuppressive microenvironment, but they also facilitate immune evasion and promote metastasis by altering the structure and function of TDLNs. Research has shown that tumor-specific memory CD8^+^ T cells (T_TSM_) within TDLNs are essential for the efficacy of immune checkpoint inhibitors, such as PD-1/PD-L1 blockers. Moreover, the abnormal structure of TDLNs, along with the presence of immunosuppressive cells—such as regulatory T cells (Tregs), regulatory B cells (Bregs), and immunosuppressive dendritic cells (DCs)—contributes to tumor-mediated immune evasion. Therefore, gaining a deeper understanding of the immune microenvironment within TDLNs is essential for improving the effectiveness of immunotherapies and developing novel therapeutic strategies. This review explores various TDLN-based therapeutic strategies, addressing the controversies surrounding lymph node dissection, the use of TDLNs as a source of tumor-infiltrating lymphocytes (TILs) for therapy, targeting immunosuppressive cells within TDLNs, and methods to reverse the structural abnormalities of TDLNs. These strategies offer valuable insights and potential directions for advancing tumor immunotherapy.

## 1 Introduction

In recent years, tumors have been recognized as highly complex systemic diseases that, in addition to their malignant proliferation, induce profound alterations across various bodily systems. These changes collectively create “sanctuaries” that shield the tumor from immune system surveillance. Specifically, tumors actively construct immunosuppressive microenvironments that significantly undermine the efficacy of CD8^+^ T cell-mediated anti-tumor responses, which are central to immune defense mechanisms (1). Currently, tumor immunotherapy strategies, particularly immune checkpoint blockade, aim to rejuvenate the anti-tumor potential of CD8^+^ T cells within the tumor microenvironment (TME) (2). This approach has achieved significant efficacy in clinical practice and has emerged as one of the most promising therapeutic strategies in oncology. However, substantial challenges remain in clinical settings: only a small percentage of patients with solid tumors respond to immunotherapy, and the majority fail to achieve durable or substantial therapeutic benefits (3).

As research into tumor immunology advances, the pivotal roles of TDLNs in immunotherapy has garnered increasing attention. It has been discovered that the effectiveness of immune checkpoint inhibitors, including PD-L1 inhibitors, not only depends on the reactivation of pre-existing exhausted CD8^+^ T cells within the TME, but also relies heavily on the continuous influx of newly activated and expanded effector T cells from the periphery, particularly within the TDLNs (4). The PD-1-PD-L1 interaction in the TDLN, rather than within the tumor itself, can serve as a predictive marker for the clinical efficacy of PD-L1 inhibitors, particularly in tumors like metastatic melanoma (5). Moreover, studies have shown that tumor antigen-specific memory CD8^+^ T cells, which are crucial responders to PD-L1 inhibitors, are predominantly located within TDLNs (6). However, as tumors progress, they can significantly suppress anti-tumor immune responses by reprogramming the TDLN microenvironment. This process includes reshaping the structural architecture of TDLNs and regulating the differentiation and function of immunosuppressive cells, which ultimately limits the efficacy of immunotherapy. Therefore, a comprehensive understanding of the mechanisms driving TDLN immune microenvironment remodeling is essential for elucidating tumor immune evasion, enhancing the efficacy of immunotherapies, and developing novel therapeutic strategies. This review summarizes the current research on TDLN immune microenvironment remodeling and its implications for immunotherapy. Additionally, we explored relevant therapeutic strategies, aiming to provide new insights and references for future cancer immunotherapy.

## 2 The crucial role of tumor-draining lymph nodes in tumor immunotherapy

### 2.1 TDLN serves as the initiating site of the anti-tumor immunity cycle

During tumor progression, the TDLN is not only serves as the primary site where tumor cells arrive via the lymphatic system but also acts as the initiation point for anti-tumor immune responses. The tumor immunity cycle is a sequential process wherein the immune system first recognizes tumor cells, activates specific T cells, and then directs them to the tumor site for elimination. The most crucial steps of antigen presentation and T cell activation occur precisely within the TDLN (7). Recent studies have shown that naive T cells, upon activation in the TDLN in the presence of tumors, do not immediately differentiate into effector cells. Instead, they enter a “stem-like” state, from which they can develop into either exhausted T cell precursors/progenitors (T_pex_ cells) or T_TSM_ (6, 8). These cells express high levels of stem cell-related markers such as TCF-1 but lack effector molecules like granzyme B and perforin (9). It is only after migrating to the tumor microenvironment and receiving additional co-stimulatory signals and cytokine stimuli that these precursor cells differentiate into fully functional effector CD8^+^ T cells, acquiring the capacity to kill tumor cells. However, numerous studies have indicated that within the TME, activated CD8^+^ T lymphocytes often lose their ability to develop into memory cells due to persistent antigen stimulation and the inhibitory effects of the immune suppressive milieu. Initially, these activated T cells differentiate into T_pex_ cells, which represent a less exhausted state, but eventually progress into terminally exhausted T cells (10–14). This process is marked by a progressive loss of effector function, proliferative capacity, and memory potential, accompanied by sustained high expression of immune checkpoint molecules, such as PD-1 and TIM-3 (15). Based on this understanding, immune checkpoint blockade (ICB) therapies, particularly targeting PD-1/PD-L1, have shown promise in partially reversing the exhaustion of CD8^+^ T cells and, consequently, controlling tumor progression.

The prevailing viewpoint within the field is that T_pex_ cells within the TME are the primary responders to PD-1/PD-L1 ICB treatment (16). However, it is important to recognize that T_pex_ cells represent a relatively small proportion of the TME, constituting only about 5% (17). Moreover, recent research has revealed that after receiving PD-1/PD-L1 ICB therapy, patients with tumors develop novel TCR clones of tumor-localized, antigen-specific CD8^+^ T cells that were previously absent (18). These findings suggest that the tumor-specific T cells responding to ICB treatment are largely dependent on the continuous replenishment of CD8^+^ T cells from outside the tumor, particularly from the TDLNs (4, 19); however, this replenishment process diminishes as the tumor progression advances. For example, in a study on head and neck cancer, T_pex_ cells in uninvolved lymph nodes (LNs) were found to undergo activation and differentiation following PD-L1 treatment, whereas these signals were impaired in lymph nodes affected by cancer metastasis (18). A study utilizing FTY720 to inhibit T cell recruitment from the TDLN to the tumor site showed a substantial effect on early tumor response (20); however, at later stages of tumor progression, this inhibition had minimal impact on tumor progression. This observation suggests that, as tumors progress, T cell activation within the TDLN may become impaired. In 2023, Professor Ye Liling’s team conducted a series of tumor transplantation and lymph node excision experiments, which clarified that PD-1/PD-L1 ICB therapy primarily mobilizes TDLN-T_TSM_ (6). These cells then expand and differentiate into T_pex_ cells, which subsequently enter the TME to exert their effects, rather than attempting to reverse the exhaustion of T_pex_ cells already infiltrated within the tumor. Furthermore, the TDLN-T_TSM_ cells play a critical role in determining the therapeutic efficacy of PD-1/PD-L1 ICB treatment. The studies highlight the significant role of TDLNs in shaping the anti-tumor immune response and influencing the response to ICB treatment. They underscore the importance of exploring the immune microenvironment of TDLNs to better understand the mechanisms underlying tumor immune evasion and resistance to immunotherapy.

### 2.2 The TDLN is a crucial structure within the lymphatic system for tumor metastasis

**Figure 1:**
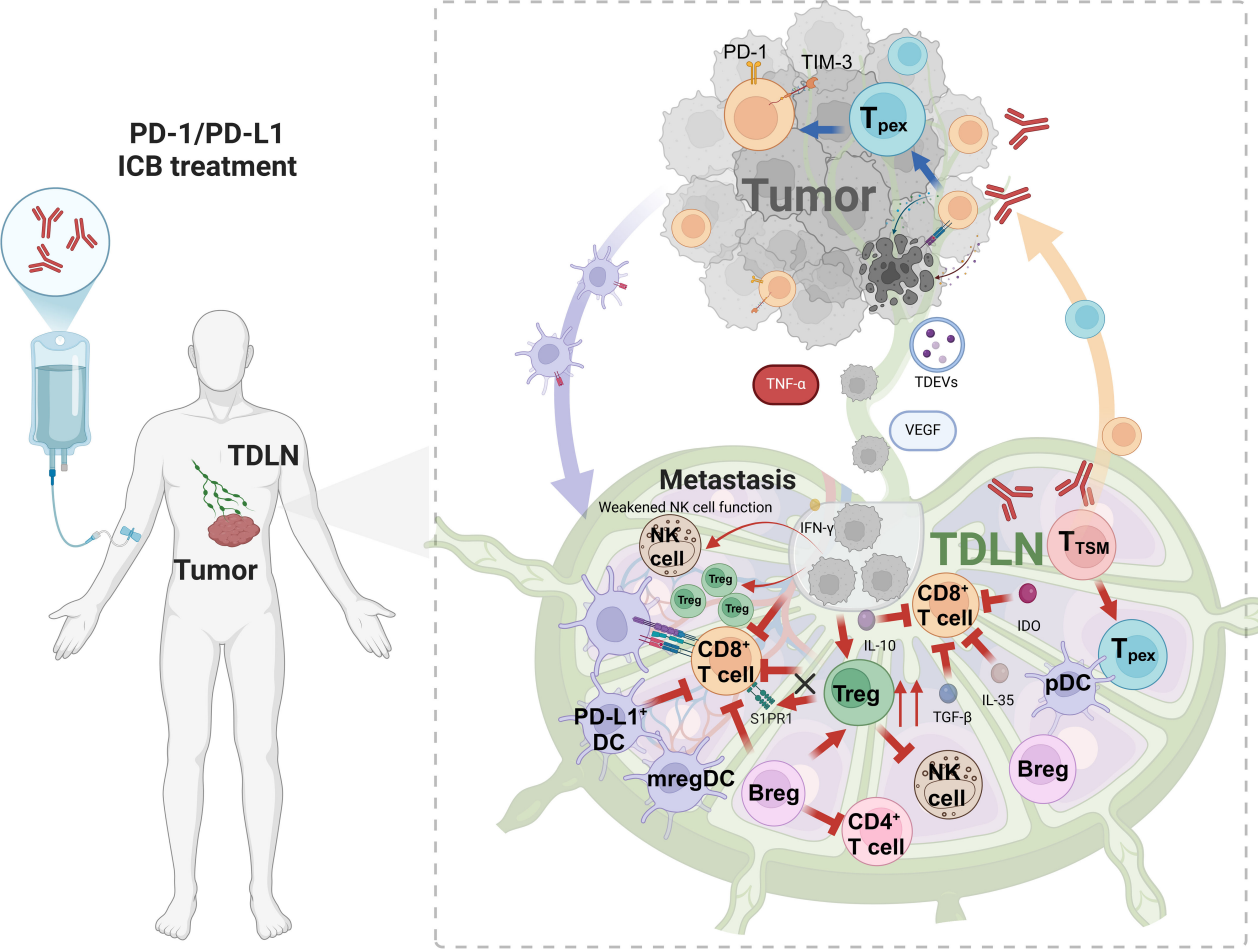
The immunosuppressive microenvironment within TDLNs. TDLN is the starting point of anti-tumor immunity and activates initial T cells. The antigen presented by DC activates CD8+ T cells (such as Tpex/T_TSM_), which migrate to tumors and differentiate into effector cells. At the same time, TDLN promotes metastasis: VEGF induces lymphangiogenesis, and immune cells construct an immunosuppressive environment. Treg(IL-10/PD-1), mregDC(IDO), and Breg(IL-10/TGF-β/IL-35) cooperate to suppress immune response, drive tumor escape, and limit treatment response. Figure was created with Biorender.com.

During tumor progression, tumors remotely regulate the immunosuppressive microenvironment within TDLNs through mechanisms such as the lymphatic transmission of inhibitory cytokines, tumor-derived extracellular vesicles (TDEVs), and other soluble factors (**Figure 1**). Additionally, tumors alter the adhesion properties within lymph nodes by upregulating metastasis-related genes and secreting lymphangiogenic factors like VEGF and extra cellular vessels (21, 22), which stimulate the formation of lyric vessels and facilitate tissue metastasis. Tumor cells can also exit lymph nodes by invading lymph node blood vessels after nodal metastasis and enter the bloodstream, thereby colonizing distant organs (23, 24). In mouse models, PD-L1 immunotherapy was found to impair the activation of CD8^+^ T cells in metastatic lymph nodes (25). Similarly, a clinical study involving 68 non-small cell lung cancer patients demonstrated that patients with metastasis to lymph nodes often exhibited poorer responses to immunotherapy (26). Consequently, when cancer spreads to the lymph nodes, selective lymph node dissection and clearance are often performed to prevent further metastasis. However, the necessity of lymph node clearance remains controversial, as it does not always lead to significant improvements in survival rates (27–30). Research on colorectal cancer shows that lymph node and distant metastases originate from independent subclones in 65% of cases, while 35% share a common origin, indicating a complex and diverse evolutionary relationship in metastasis. This diversity may explain the limitations of lymph node dissection effectiveness (31).

The TDLNs represent both a critical site for anti-tumor immune responses and a key location for tumor metastasis, making their microenvironment exceptionally complex. Nevertheless, current research has predominantly focused on the immunosuppressive microenvironment of tumors, leaving a significant gap in our understanding of the immune landscape within TDLNs.

## 3 Factors of TDLN immune tolerance

As tumors progress, significant alterations occur in both the anatomical structure and cellular composition of TDLNs. These alterations directly or indirectly impair immune cell functions, enabling tumors to evade immune surveillance and continue their growth and dissemination. Gaining a deeper understanding of these structural modifications is crucial for enhancing the efficacy of tumor immunotherapy.

### 3.1 Immunosuppression mediated by TDLN structural remodeling

In the early stages of tumor development, tumors disrupt or remodel the lymph node structure at distant sites, fostering the creation of an immunosuppressive microenvironment within TDLNs and laying the groundwork for tumor metastasis. It has been observed in animal models that tumor cells influence this process by regulating factors such as integrin αIIb and VEGF, which promote extensive lymphangiogenesis within the TDLNs which results in the dilation of lymphatic sinuses and the rapid proliferation of lymphatic endothelial cells (22, 32–34). Although recent research of this phenomenon is limited in human cancer models, it has undoubtably been observed in TDLNs of patients (35–37). Consequently, the increased flow of lymphatic fluid enhances the accumulation of tumor-derived factors in the TDLNs, further suppressing the local immune environment and accelerating tumor metastasis to these lymph nodes (38). Additionally, the expansion and dedifferentiation of high endothelial venules (HEVs) represent key features of tumor-induced lymph node remodeling. Over the course of tumor progression, the density of HEVs increases, followed by their gradual expansion and dedifferentiation, which significantly affect lymphocyte recruitment (39, 40). However, After tumor colonization in TDLNs, tumor cells cause HEV numbers to drop, which limits lymphocyte recruitment into the colonized node (41). Moreover, fibroblastic reticular cells (FRCs) in the TDLN contribute to the deposition of the extracellular matrix components, enhancing their proliferative capacity and driving fibrotic remodeling of the surrounding ductal structures. This remodeling alters the structural flexibility of the lymph nodes, which impacts immune cell trafficking and function (42).

### 3.2 The immunosuppressive cells in TDLNs

#### 3.2.1 Tumor immunosuppression mediated by Treg cells in TDLN

A subset of CD4^+^ T cells, known as regulatory T cells (Tregs) (43, 44), exhibits potent immunosuppressive properties, which contribute to tumor immune evasion. Within the TME, Tregs suppress the immune response against tumors through both direct intercellular interactions and the release of soluble cytokines, thereby playing a critical role in enabling tumors to evade immune surveillance. During tumor progression, tumor-derived signals and lymph node colonization by tumor cells direct the accumulation of Tregs in TDLNs (45–48). It has been observed that Tregs infiltrate TDLNs in significant numbers during cancer progression, and their accumulation often correlates with poorer clinical outcomes (49). Additionally, the Tregs in TDLNs exhibit enhanced immunosuppressive functions, such as upregulated expression of co-inhibitory molecules (e.g., CTLA-4 and PD-1) and increased secretion of immunosuppressive cytokines, including TGF-β and IL-10 (50) (**Figure 1**). Tregs in TDLNs also hinder the migration of activated CD8^+^ T cells to the tumor site by downregulating sphingosine-1-phosphate receptor 1 (S1PR1) on these lymphocytes (**Figure 1**). This impairment in CD8^+^ T cell trafficking limits their cytotoxic potential and further suppresses the anti-tumor immune response. Moreover, Tregs have been shown to inhibit the function of natural killer (NK) cells in TDLNs, thereby promoting tumor metastasis (51). In TDLNs, Tregs also uptake lactate through the high expression of monocarboxylate transporter 1 (MCT1) (52), which enhances PD-1 expression on Tregs. This mechanism contributes to resistance to PD-1 blockade therapy. Interestingly, while depleting Tregs could disrupt TDLN function and expand the T cell zone, it might also indirectly influence T cell recruitment and disrupt immune surveillance-stroma interactions, potentially accelerating tumor progression (53).

#### 3.2.2 Tumor immunosuppression induced by DC subsets in TDLNs

Dendritic cells (DCs), as professional antigen-presenting cells, are central to orchestrating the anti-tumor immune response. DCs capture, process, and present tumor antigens, express the major histocompatibility complex molecules, and provide co-stimulatory signals essential for T cell activation. In TDLNs, DCs also activate naive T cells, initiating the immune response against tumors. However, during tumor progression, DCs undergo a functional shift from immune activation to immune suppression (54–56). The ability of DCs to activate immune responses or induce immune tolerance in TDLNs mainly depends on their activation status (57). In the tumor milieu, insufficient activation of DCs results in ineffective antigen presentation, preventing the proper activation of T cells against tumors. Moreover, tumor-derived signals, such as type II interferons, induce the expression of PD-L1 on DCs, which then migrate to TDLNs. These PD-L1-expressing DCs, predominantly located in the germinal centers and cortical regions of TDLNs, suppress the activation of CD8^+^ T cells through the PD-1/PD-L1 interaction (20) (**Figure 1**). Studies have shown that the accumulation of PD-L1^+^ conventional DC1 (cDC1) cells in TDLNs is associated with an increase in terminally exhausted CD8^+^ T cells, as well as an elevated risk of metastasis or disease recurrence in patients with ovarian cancer, oral squamous cell carcinoma, and lung metastatic melanoma (20, 58). Abundant PD-1/PD-L1 interactions of PD-1^+^ T cells and PD-L1^+^ cDCs in TDLNs has also been linked to poor survival and distant disease recurrence in cancer patients (4). Single-cell analysis has identified a subset of mature DCs, termed regulatory DCs (mregDCs), which accumulate in TDLNs during tumor progression. These mregDCs express immune regulatory genes such as Cd274, Cd200,and Pdcd1lg2, along with mature genes such as Ccr7,Cd40, and Il12b (59). These cells enhance their migratory capacity and migrate to TDLNs, while simultaneously downregulating the expression of toll-like receptors (TLRs) and lectin-like receptors, which impairs their ability to capture and present antigens. Additionally, mregDCs secrete chemokines and express adhesion molecules that attract Treg to form a distinct Treg-mregDC-lymphatic niche. This network further inhibits the initiation and maintenance of the anti-tumor immune response through the secretion of immunosuppressive cytokines and the expression of immune checkpoint molecules (60).

Despite representing a small proportion of the total DC population, mregDCs exert potent immunosuppressive effects through both direct cell-to-cell interactions and the secretion of immune regulatory factors. These cells suppress the antigen-presenting function of neighboring DCs, inhibit CD8^+^ T cell responses, and promote the differentiation of Tregs and Th2 cells (61). Some studies have also identified plasmacytoid DCs expressing indoleamine 2,3-dioxygenase (IDO) in TDLNs (**Figure 1**), which directly activate Treg cells to enhance immunosuppressive functions (62–65). Furthermore, tumor cells can inhibit tumor immunity not only by affecting the function of DCs but also by altering their quantity (66). Thus, the role of DCs in TDLNs is complex and highly heterogeneous, with tumor-derived factors driving the functional plasticity of these cells. Identifying strategies to specifically eliminate or correct immunosuppressive DC populations in TDLNs remains a significant challenge in the field of cancer immunotherapy.

#### 3.2.3 Tumor immunosuppression induced by Bregs within the TDLNs

In recent years, regulatory B cells (Bregs), a novel subset of B cell, have garnered increasing recognition for their role in immune regulation. Bregs primarily mediate immune tolerance by generating inhibitory cytokines such as TGF-β and IL-10, which suppress excessive inflammatory responses (67–69). Bregs are crucial in the outcome of chronic inflammatory diseases, and recent studies have highlighted their importance in the TME. In tumors such as gastric cancer, pancreatic cancer, and lung cancer, Breg cells are found to increase abnormally as the tumor progresses (70, 71). Moreover, Bregs exert their immunosuppressive effects primarily by inhibiting the functions of CD4^+^ T and CD8^+^ T cells by secreting IL-10, IL-35, and TGF-β (**Figure 1**). And promote the differentiation and proliferation of Treg cells, further contributing to immune evasion by the tumor (21, 72, 73). Notably, Bregs accumulate in TDLNs, where they play a significant role in promoting tumor growth (74–76). Bregs hinder the synthesis of IL-17 and IFN-γ by Th17 and Th1 cells, respectively, and prevent the differentiation of Th17 cells within the tumor microenvironment in an IL-10-dependent manner (77, 78). Furthermore, Bregs in TDLNs are more prone to differentiate and exert immunosuppressive effects, secreting cytokines such as TGF-β and IL-35 (72, 73). These findings underscore the critical role of Bregs in suppressing immune surveillance and promoting metastasis within the TDLN by modulating immune responses and enhancing immunosuppression in the tumor microenvironment.

### 3.3 Immune suppression mediated by tumor cells in TDLNs

Tumor colonization in lymph nodes induces tumor immune tolerance and further promotes distant metastasis (47). Numerous studies have shown that the primary tumor plays a preparatory role in establishing an immunosuppressive microenvironment within TDLNs to facilitate subsequent lymph node metastasis through the secretion of vesicles, cytokines, and other factors (79–82). One key mechanism involves the activation of lymphangiogenesis via VEGF, which alters the lymphatic matrix in the TDLN (83). Additionally, (**Figure 1**) TNF-α plays a crucial role in promoting inflammatory reactions within the TDLN by binding to TNF receptors (TNFR1 and TNFR2) on cell surfaces. This interaction activates downstream signaling pathways, leading to the production of inflammatory mediators such as IL-1, IL-6, and IL-8, which inhibit immune cell recruitment to the TDLN (84). Furthermore, tumor cells can remodel the TDLN into an immunosuppressive microenvironment that supports metastasis by secreting immunosuppressive molecules within exosomes. These exosomes, including melanoma-derived exosomes carrying PD-L1, shuttle tumor-derived signals to TDLNs, thereby promoting immune evasion. For example, melanoma-derived exosomes enhance PD-L1 expression on immature myeloid cells (IMCs) in mice, resulting in the inhibition of T cell activation. Interferon-gamma (IFN-γ) further augments PD-L1 expression on these exosomes, further impairing immune responses (81, 85). Once tumor cells metastasize to the lymph nodes, they directly influence the TDLN microenvironment. Tumor cells establish a hypoxic and acidic environment, accompanied by the release of large quantities of immunosuppressive cytokines such as IL-10 and TGF-β. This milieu not only promotes the expansion and activation of Tregs but also induces immune cells in the TDLN to adopt an immunosuppressive phenotype. As a result, the immunological function of the lymph nodes is suppressed, enabling the tumor to evade immune surveillance more effectively (86, 87).

## 4 Tumor treatment strategies based on TDLNs and prospects

### 4.1 Controversy surrounding lymph node dissection

**Figure 2:**
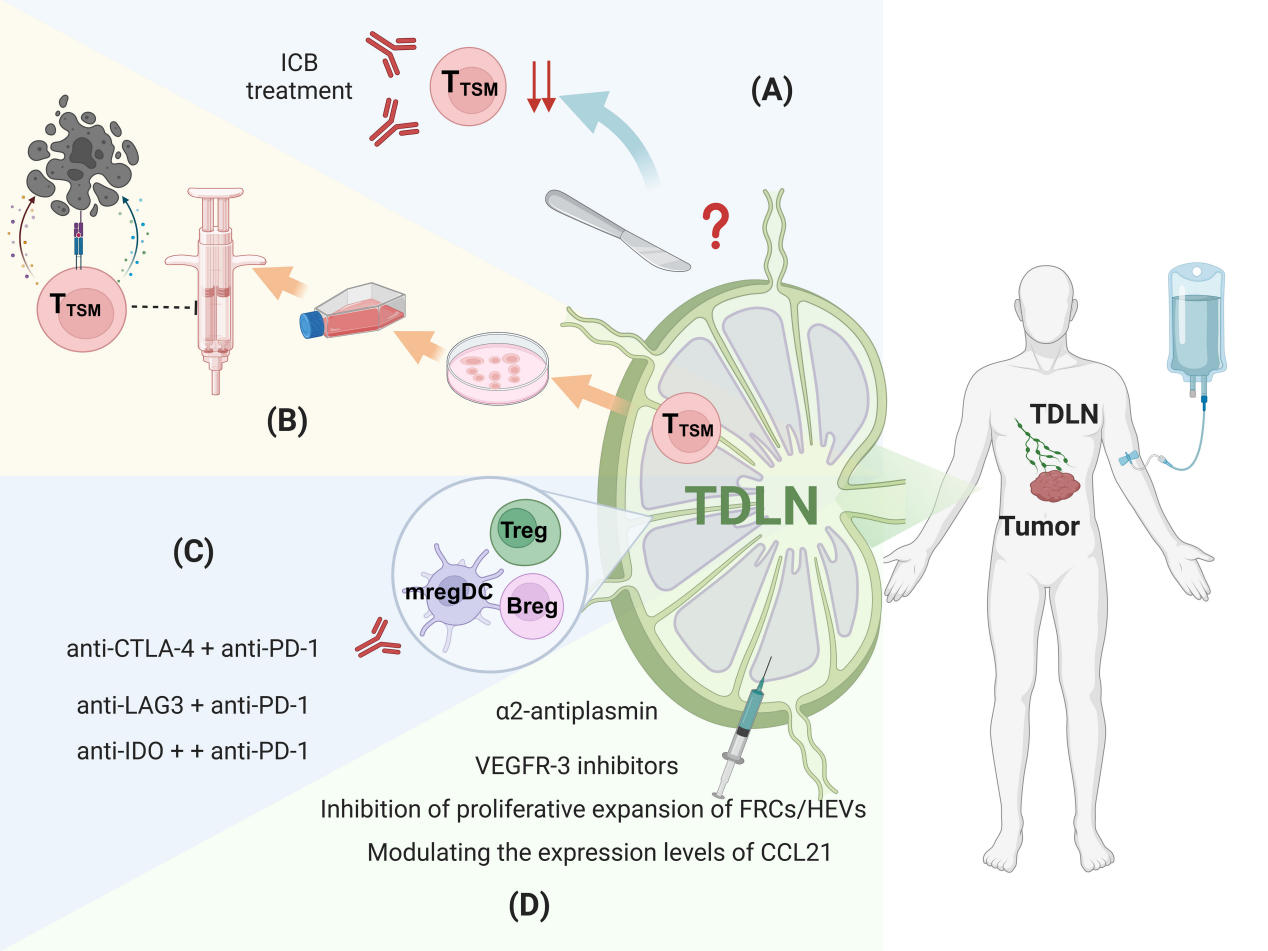
Therapeutic strategies targeting the immunosuppressive microenvironment of TDLNs. **(A)** Lymph Node dissection, **(B)** Seed cells for ACT therapy, **(C)** Targeting the immunosuppressive cells, **(D)** Reversing abnormal structure. Figure was created with Biorender.com.

Lymph node dissection is widely recognized as an essential procedure for reducing tumor burden and lowering the risk of distant metastasis (88–90) (**Figure 2**). Currently, lymph node dissection is classified into selective, systematic, or radical clearance. The prevailing view suggests that systematic or radical dissection provides a better prognosis compared to selective dissection. A 15-year follow-up study found that the gastric cancer-related mortality rate in the D1 group (local lymph node dissection) was significantly higher than in the D2 group (radical lymph node dissection) (91). Similarly, in thymic neuroendocrine tumors, radical lymph node dissection has been identified as the best treatment option (92). In non-small cell lung cancer (NSCLC), lymph node dissection is associated with a lower risk ratio compared to local biopsy or excision (29). However, with the growing understanding of the role of TDLNs, the practice of lymph node dissection has become increasingly controversial. For instance, a clinical study on esophageal cancer found that patients undergoing extensive lymph node dissection did not exhibit significantly better five-year overall survival rates, suggesting that more aggressive lymph node clearance may not necessarily improve prognosis (93, 94). Similarly, a study on recurrent non-small cell lung cancer patients, who were grouped based on the number of dissected lymph nodes (using a cutoff of 16), revealed that patients with more than 16 lymph nodes dissected showed poorer responses to immunotherapy (95). In endometrial cancer, the prognosis was found to be unaffected by pelvic lymphadenectomy (96). Notably, a clinical study on stage I-II ovarian malignant germ cell neoplasms demonstrated that the 10-year disease-free survival rate was greater in the group that does not undergo lymph node dissection compared to the group that did (97). Likewise, in melanoma, immediate completion lymph-node dissection after sentinel-node metastasis did not increase melanoma-specific survival compared to observation (98). In breast cancer, sentinel lymph node dissection alone was non-inferior to axillary lymph node dissection for 10-year overall survival in certain patients (99). These findings suggest that the traditional ‘‘one-size-fits-all’’ approach to lymph node dissection may not be optimal. TDLNs not only serve as a pre-metastatic niche for tumors but also play a critical role in initiating the anti-tumor immune response. As such, lymph node dissection may inadvertently disrupt key immune events within the TDLNs and eliminate crucial cell populations essential for an effective immunotherapy response. Therefore, considering the functional diversity, cellular heterogeneity, and dynamic plasticity of TDLNs driven by tumor evolution, more comprehensive and systematic studies are required to guide clinical decisions regarding lymph node dissection.

### 4.2 TDLNs as potential sources of seed cells for adoptive cell therapy

Adoptive cell therapy(ACT), a form of adoptive cell therapy, has emerged as a promising cancer treatment in recent years (100). This therapy involves isolating T lymphocytes that specifically recognize tumor antigens from the patient, expanding them ex vivo, and reinfusing them to target and eliminate tumor cells. Currently, peripheral blood or tumor tissue serves as the primary source of ACTs. However, these T cells often exhibit exhaustion due to prolonged exposure to the immunosuppressive TME, resulting in suboptimal therapeutic outcomes and limited durability. The discovery of T_TSM_ within the TDLN, which demonstrate enhanced proliferative capacity and reduced exhaustion, offers a promising alternative source of ACTs. T_TSM_ cells derived from TDLN exhibit remarkable anti-tumor activity in animal models and are more effective in combination with PD-1/PD-L1 ICB therapy (6). This indicates that T cells from TDLNs could serve as superior seed cells for anti-tumor adoptive T cell therapy, providing new insights and considerations for future ACT treatment strategies (**Figure 2**). Chimeric Antigen Receptor T-cell (CAR-T) therapy is also a highly anticipated treatment approach. However, its current limitations include the limited selection of tumor targets, off-target toxicity, and relatively poor efficacy in solid tumors (101–104). Given that T cells in TDLNs are T cells with strong tumor specificity, utilizing them for CAR-T therapy may offer greater potential than conventional CAR-T treatments. However, research in this area is still in its early stages, and experimental evidence is yet to be established.

### 4.3 Targeting the immunosuppressive cells in TDLNs

Immunosuppressive immune cells, such as Tregs, Bregs, and mregDCs, accumulate in TDLNs and contribute to the establishment of an immunosuppressive microenvironment. Targeting these immunosuppressive cells in both the tumor site and TDLNs could lead to more favorable treatment outcomes.

Animal models have demonstrated that combining CTLA-4 and PD-L1blockade results in a favorable shift in the balance of effector T cells and Tregs within the local tumor microenvironment, showing greater efficacy than monotherapy with either blocker (105). Furthermore, studies have indicated that combined anti-CTLA-4 and anti-PD-1 therapy induces a durable anti-tumor response and enhances adoptive cell therapy in mice (106). Clinical trials also support these findings. For example, a phase III trial involving 945 patients with unresectable stage III and IV melanoma demonstrated that patients receiving the combination of ipilimumab (anti-CTLA-4) and nivolumab (anti-PD-1) had longer median progression-free survival compared to those receiving either therapy alone (107). Additionally, the combination of nivolumab with relatlimab (anti-LAG3) was approved by the FDA as first-line treatment for stage III/IV melanoma due to its progression-free survival benefit (108). In the TDLN, mregDCs express immune checkpoints such as IDO and PD-L1, making IDO an attractive therapeutic target. In a clinical trial, the combination of nivolumab with a vaccine targeting immune cells expressing IDO and PD-L1 induced immune responses in more than 93% of patients (109). The vaccine-responsive T cells, including CD4^+^ and CD8^+^ T cells, were able to target both cancer cells and immune cells expressing these markers. Follow-up studies showed sustained and significant therapeutic effects in some patients (110) (**Figure 2**). These findings suggest that targeting the immunosuppressive microenvironment in TDLNs in combination with local tumor treatments, such as PD-1/PD-L1 blockade, may improve cancer treatment outcomes. In addition to combination ICB, there are other approaches to targeting immunosuppressive cells. Recent studies have shown that delivering antigens to the interfollicular regions (IFRs) of lymph nodes using specific immunization formulations can enhance Type 1 immune responses (Th1 responses), potentially providing a complementary strategy to reverse immunosuppression in TDLNs (111). Moreover, targeted delivery of adjuvant nanoparticles, vaccine antigens, and other molecules to TDLNs can also help improve the immunosuppressive microenvironment (112, 113).

### 4.4 Reversing abnormal structural changes in TDLNs

Several therapeutic strategies aim to reverse the structural changes in TDLNs caused by tumor derived factors, which contribute to immunosuppression. These strategies focus on inhibiting tumor-induced lymph node remodeling and angiogenesis. For example, the fibrinolytic inhibitor α2-antiplasmin has been shown to restrict lymph node remodeling and cancer metastasis by inhibiting fibrin degradation, thereby preventing lymphatic dilation and abnormal changes (114). In addition, VEGFR-3 inhibitors have been verified to reduce the proliferation of lymphatic endothelial cells (LECs), further impeding vascular remodeling and lymphangiogenesis (115). Targeted therapies that address lymph node structural remodeling can also restore immune cell distribution by blocking the signaling pathways involved in immune cell migration. Inhibiting of FRCs and HEVs can prevent the formation of the immunosuppressive microenvironment by regulating immune cell migration and positioning (116). Of these, treatment of FRCs and HEVs has focused on reducing stress and alleviating tumor-induced impaired lymphocyte recruitment. In mice, treatment with losartan, is an angiotensin receptor blocker that relieves solid stress, reduces collagen in LN metastatic lesions, Restores the presence of different HEV, and beautiful intrinsic lyric infiltration (41). Furthermore, modulating the expression levels of chemokines, such as CCL21, can improve immune cell distribution and function, thereby restoring normal TDLN structure. Interventions targeting physical changes, such as reducing FRC proliferation and abnormal extracellular matrix (ECM) deposition, can alleviate the immunosuppressive state in TDLNs, promoting the recovery of anti-tumor immune responses (**Figure 2**).

## 5 Conclusion and discussion

TDLNs serve as critical crossroads in cancer, orchestrating anti-tumor immune responses while simultaneously being exploited by tumors to foster immunosuppression and metastasis. These nodes are essential for initiating immunity, particularly through T cells that underpin the efficacy of therapies like PD-1/PD-L1 blockade. Yet, tumors disrupt this role by inducing structural remodeling—such as lymphangiogenesis, and recruiting immunosuppressive cells, including Tregs, Bregs, and immunosuppressive DC subtypes, creating a suppressive microenvironment that hampers immune function. Emerging therapeutic strategies offer promise, such as leveraging TDLN-derived T cells for adoptive cell therapy and targeting immunosuppressive pathways to enhance immunotherapy outcomes. However, the TDLN’s complexity and dynamic nature present challenges, including difficulties in drug delivery and the need for reliable biomarkers to predict responses. Advancing these approaches will require a deeper understanding of TDLN plasticity and the exploration of combination therapies to synergistically improve cancer treatment efficacy.
